# Feasibility of 7-T fluorine magnetic resonance spectroscopic imaging (^19^F MRSI) for TAS-102 metabolite detection in the liver of patients with metastatic colorectal cancer

**DOI:** 10.1186/s41747-018-0043-8

**Published:** 2018-07-11

**Authors:** Sophie A. Kurk, Bart R. Steensma, Anne M. May, Miriam Koopman, Hans M. Hoogduin, Tijl A. van der Velden, Dennis W. J. Klomp, Wybe J. M. van der Kemp

**Affiliations:** 1 Department of Medical Oncology, University Medical Center Utrecht, Utrecht University, Heidelberglaan 100, 3584 CX Utrecht, the Netherlands; 2 Julius Center for Health Sciences and Primary Care, University Medical Center Utrecht, Utrecht University, Heidelberglaan 100, 3584 CX Utrecht, the Netherlands; 3 Department of Radiology, University Medical Center Utrecht, Utrecht University, Heidelberglaan 100, 3584 CX Utrecht, the Netherlands

**Keywords:** Biomarkers, Colorectal neoplasms, Magnetic resonance spectroscopy (MRS), Metabolism, Trifluridine/tipiracil (TAS-102)

## Abstract

Trifluridine/tipiracil (TAS-102) has shown a significant overall survival benefit in patients with heavily pre-treated metastatic colorectal cancer. However, predicting treatment response and toxicity in individual patients remains challenging. Fluorine (^19^F)-containing drugs can be detected with magnetic resonance spectroscopy (MRS) to determine the metabolic rates and the biodistribution of the drug in normal and tumour tissue, which are related to treatment efficacy and toxicity. This is the first study to investigate the potential of 7-T ^19^F-MRS to detect TAS-102 metabolites in humans. We demonstrate that, with the used setup, TAS-102 is not detectable in liver metastases of metastatic colorectal cancer patients on a normal treatment schedule. Therefore, ^19^F-MRS TAS-102 metabolite detection is not yet useful for the clinical early prediction of treatment response. As ^19^F-MRS is able to detect TAS-102 in phantom and murine models, the use of ^19^F-MRS remains a potential tool to noninvasively detect and possibly monitor the metabolism when higher dosages of TAS-102 are administered, for example in organoid and animal studies.

## Key points

- Predictive biomarkers for TAS-102 efficacy could enable personalisation of colorectal cancer treatment.
- ^19^F-MRS enables to noninvasively monitor the metabolism of fluorinated drugs.
- TAS-102 was detected in a phantom model with ^19^F-MRSI.
- TAS-102 was not detected with ^19^F-MRSI in humans on standard treatment.

## Background

Trifluridine/tipiracil (TAS-102), a combination of the nucleoside trifluridine (TFT) and the phosphorylase inhibitor tipiracil hydrochloride, is a novel oral fluorinated chemotherapeutic agent [1]. Recently, TAS-102 has become available for metastatic colorectal cancer (mCRC) treatment after demonstrating a significant survival benefit in patients who are refractory/intolerant to all standard mCRC therapies [2, 3]. During standard TAS-102 treatment, patients are treated for approximately eight weeks before the efficacy of the treatment can be determined by volumetric changes of the tumour and/or metastases on computed tomography scans. This implies that unnecessary toxicity and costs for nonresponding patients can be prevented if biomarkers could predict treatment efficacy and toxicity earlier during treatment.

Fluorine (^19^F)-containing drugs can be detected with magnetic resonance spectroscopy (MRS) to determine the metabolic rates and the biodistribution in normal and tumour tissue, which are related to treatment efficacy and toxicity and could be used to personalise treatment [4–6]. For example, previous studies used ^19^F-MRS to monitor the metabolism of the frequently used fluorinated chemotherapeutic agent 5-fluorouracil (5-FU) in the liver of patients with mCRC [7, 8]. These studies found a correlation between the maximum levels of 5-FU (anabolites) and the response to treatment. Studies also found levels of α-fluoro-β-alanine (FBAL) and α-fluoro-ureidopropionic acid (FUPA), which are known to be toxic waste products of 5-FU conversion and may be used to identify personalised toxicity [4, 9]. Over the last years, a paradigm shift has led to the development of oral (pro-)drugs (i.e. capecitabine) instead of intravenous equivalents because of their easy administration. Unfortunately, oral administration increases the difficulty of ^19^F-MRS drug detection due to lower concentrations in the body and gradual conversion of metabolites. As a consequence, predicting treatment response with MRS remains challenging*.* Yet, by using more sensitive MRI systems (with a stronger magnetic field and better detector arrays), studies have shown that metabolic conversion products of capecitabine, such as 5′-deoxy-5-fluorouridine, 5’deoxy-5-fluorocytidine, FBAL and FUPA in human liver can be detected by ^19^F-MRS and may be used to investigate personalised dose delivery [4, 9, 10].

TAS-102 shows a great potential for ^19^F-MRS imaging (MRSI) since TFT and its metabolites contain three ^19^F atoms compared to only one ^19^F atom as is the case for most fluorinated chemotherapeutic agents, including capecitabine/5-FU. Therefore, the intrinsic sensitivity for detecting TAS-102 with MRSI could be greatly improved as compared to capecitabine. When comparable concentrations in human tissues are reached, the MRS sensitivity for TAS-102 metabolite detection is likely to increase.

We present here the first study investigating the potential to detect TAS-102 metabolites in liver metastases with ultra-high field 7-T MRS equipped with an array of eight broadband fractionated dipole antennas [11].

## Methods

### Patients

This single-centre feasibility study was performed at the University Medical Center Utrecht, The Netherlands. The study protocol was considered as a protocol development study, and as such it was approved by the medical ethics committee of the University Medical Center of Utrecht. All patients gave written informed consent prior to study participation*.*

Patients were recruited from the outpatient clinic of the Department of Medical Oncology. Patients were eligible for participation if they were aged ≥ 18 years, diagnosed with mCRC with ≥ 1 liver metastases ≥ 2 cm in diameter and registered to receive TAS-102 in the compassionate use program in The Netherlands. The eligibility criteria for this program were previously described [12]. Patients were scheduled to receive 35 mg/m^2^ TAS-102 twice a day, following a 28-day cycle according to routine clinical practice [2]. Patients with a contraindication to undergo MRS examinations were excluded. For this feasibility study we aimed to enrol five patients. Given that, in a previous 7-T ^19^F- MRS study on the metabolism of capecitabine [10], capecitabine and/or its metabolites were detected in all examinations in the two patients enrolled, we assumed that the inclusion of five patients was enough for this first exploration of TAS-102 metabolite detection.

### Experimental setup

**Fig. 1:**
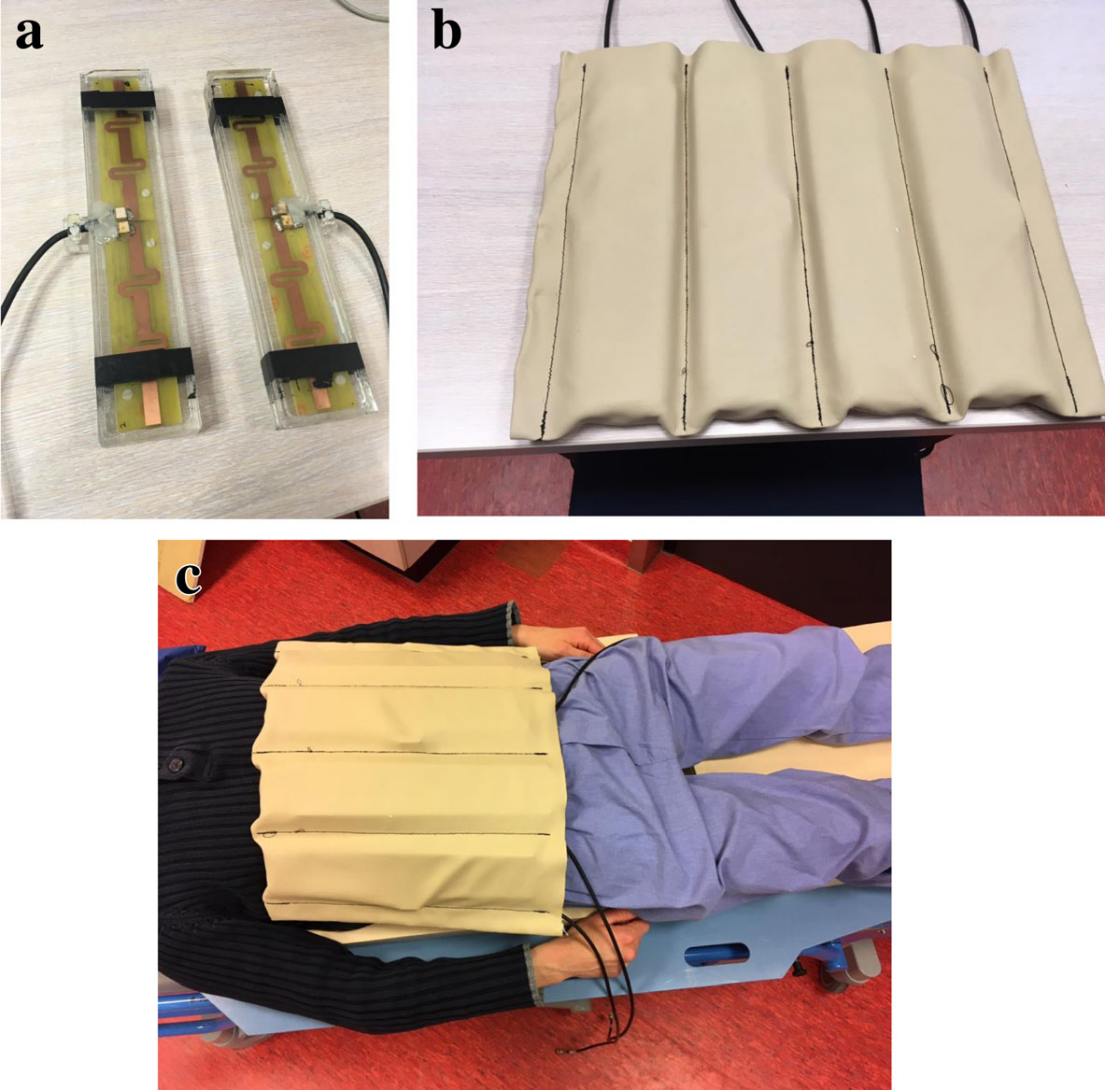
Antennas. Two broadband fractionated dipole antennas (**a**). All eight antennas (**b**). All eight antennas positioned on a volunteer (**c**). Each antenna is connected to an individual resonance frequency amplifier, enabling eight-channel B_1_ shimming

Experiments were performed on a 7-T whole body MR system (Philips, Best, The Netherlands). We used the setup described by Van Gorp et al. [10], which was previously used for MRSI of the fluorinated agent capecitabine and metabolites in the liver. In this setup, eight fractionated dipole antennas (MR Coils, Drunen, The Netherlands) were connected to the system for resonance frequency excitation and reception (Fig. 1). The coil sensitivity loss for ^19^F as compared to ^1^H is on average 7% for B_1_^−^ and 5% for B_1_^+^ [10].

Phantom measurements were performed on a 25-mL tube containing TAS-102 (corresponding to 5.4 mM TFT) positioned in a body phantom together with a 25-ml tube containing 1 M NaF for chemical shift reference, at a room temperature of 18–20 °C.

Patient measurements were performed in the morning, during cycles 1–2 of TAS-102, between days 8–12 of the cycle and 30–180 min after the oral intake of TAS-102. These time points were chosen based on previously published data on the maximum concentrations of TAS-102 and metabolites in human plasma and because previous animal studies detected TAS-102 metabolites in different tissues (including the liver and tumour) 30–120 min after TAS-102 administration [13–16]. Since food intake influences the absorption and bioavailability of TAS-102, patients were instructed to take a light breakfast in the morning prior to the MRS exam to increase the maximum concentrations of TAS-102 in the body [17]. All patients wore MR-compatible clothing. The performance of the setup was regularly tested on a TFT phantom, while during each patient measurement a small NaF tube was included for chemical shift referencing, thus ensuring proper functioning of the setup.

A similar scan protocol was used for the phantom and patient measurements. B_0_ and B_1_ shimming were performed at the ^1^H frequency before switching to the ^19^F frequency. Spoiled gradient-echo images were acquired for every single transmit channel for B_1_ calibration (radiofrequency (RF) shimming) [18]. A Matlab-based minimisation procedure (Fminsearch, Matlab, Mathworks, Natick, MA, USA) was used to determine the offset phases for the separate transmit channels, in order to maximise the average signal in a region of interest delineating the tumour. A reference scan on ^1^H was performed to enable sensitivity-weighted combination of the eight receive channels. Three-dimensional (3D) chemical shift imaging (CSI) on ^1^H was done to enable the B_1_ correction for quantification. The region of interest was positioned on the largest in diameter, non-necrotic liver metastasis, aiming at encompassing as much of the substituted parenchyma as possible. In patients with more than one metastatic lesion, optimal B_0_ shimming was performed on the largest metastasis. Phase encoding was applied along three axes for the spatial localisation of a 3D volume surrounding the liver metastases. RF shimming was used to maximise the average B_1_^+^ signal in this region. Two subsequent ^19^F 3D CSI scans were performed (one with a repetition time of 25 ms and 44 excitations, another with a repetition time of 50 ms and 22 excitations) with the following technical parameters: bandwidth of 64,000 Hz; 1024 samples; number of voxels 10 × 10 × 10; voxel size 37.6 × 37.6 × 37.6 mm^3^; a block pulse with a flip angle of 15° with a B_1_ of 12 μT on the resonance for TFT. The acquisition delay due to the excitation RF pulse and phase encoding gradients was 0.44 ms. The total scanning time for each CSI examination was 14.24 min.

### Post-processing

The data were apodised with a 50-Hz Lorentzian filter and a 50-Hz Gaussian filter in the time domain; Hamming filtering was applied in the spatial domain. We used a 1.5-mL phantom filled with 1 M NaF solution for frequency referencing at −45.9 ppm; thus, the TAS-102 metabolites of interest ranged between −1 to +13 ppm [14]. The signal-to-noise ratio (SNR) was automatically reported with custom-built interactive data language (IDL) software (Research Systems, Boulder, CO, USA). Considering the large number of data points that were obtained from the patients (three spatial dimensions and one spectral dimension), an SNR threshold ≥ 5.5 was used for the minimum detection limit of TFT and metabolites. This SNR was chosen at the 99.5% confidence interval, based purely on noise statistics, and an extended chemical shift range of possible metabolites between −8 and +20 ppm [10, 19]. An SNR of 5.5 implies a chance of 1 in $\frac{1}{1-\operatorname{erf}\left(\frac{5.5}{\sqrt{2}}\right)}$ = 26330254 of a false positive signal. A complete ^19^F CSI dataset of one patient has 1024 frequency data points per voxel, of which 12% are located within the range from −8 to +20 ppm at the acquisition bandwidth used. With a total number of voxels of 10 × 10 ×10, this amounts to 122,880 signals, implying a 0.5% chance of a false positive signal per patient. We searched for any significant SNR in all patient examinations and all liver metastases depicted within the examinations.

Additionally we performed a second, more in-depth analysis, in which we focused on a smaller chemical shift range from 5 to 13 ppm. This chemical shift range was chosen based on previously reported chemical shifts for TFT and metabolites in the liver of rats [16]. We searched for spectra with signals above the SNR threshold of ≥ 4.8 (chosen at the 95% confidence interval).

### Statistics

Continuous normally distributed variables were reported as mean ± standard deviation, while not normally distributed variables were reported as median and range.

## Results

### Phantom measurement

**Fig. 2:**
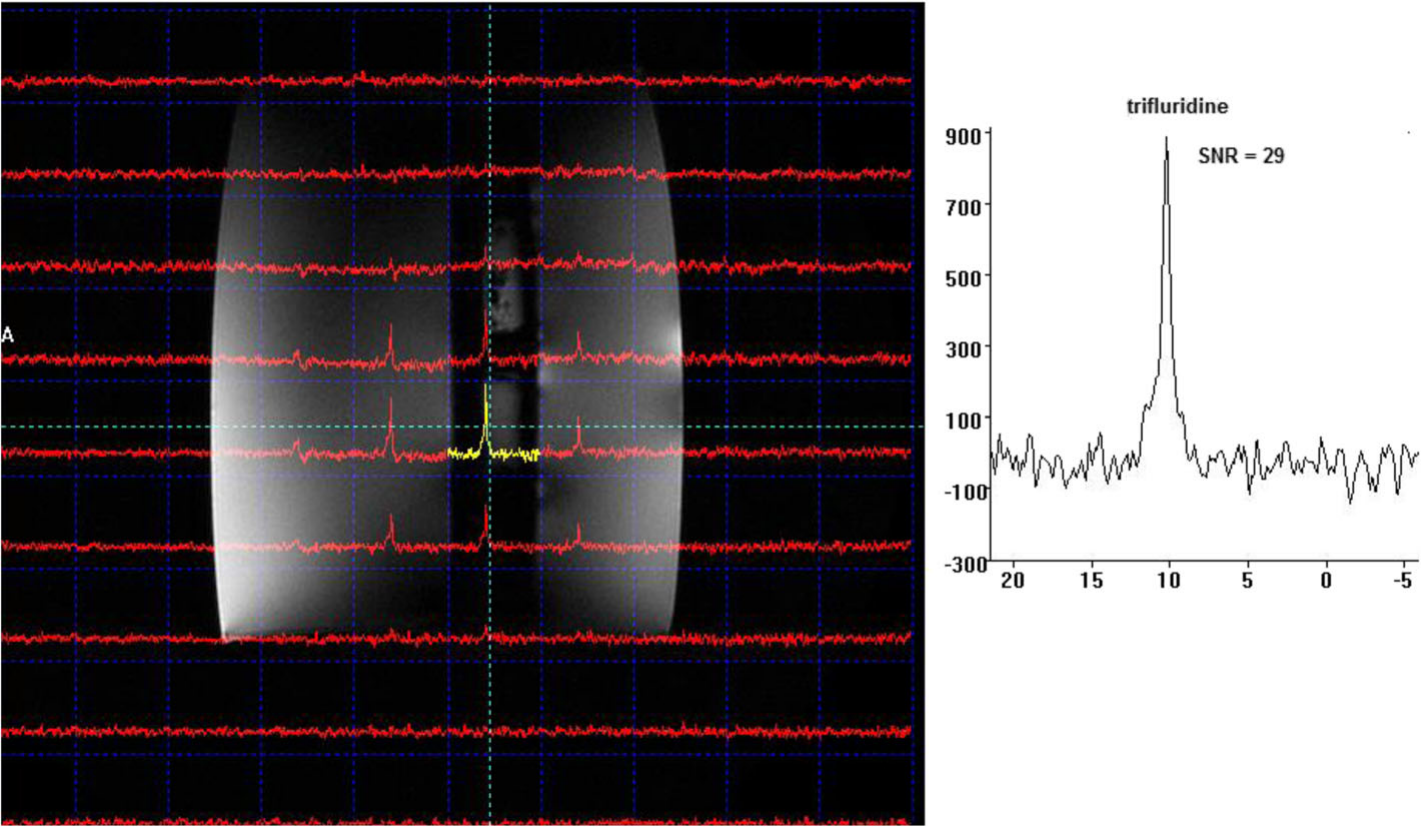
Phantom measurement. ^19^F-MRS three-dimensional chemical shift imaging scan of a 25-mL tube filled with 5.4 mM trifluridine (repetition time 25 ms; bandwidth 64,000 Hz; 1024 samples; flip angle 15°; matrix 10 × 10 × 10; field of view 376 × 376 × 376 mm. The maximum observed SNR ratio was 29. The second tube is filled with NaF used for frequency referencing (spectrum of NaF not shown)

The results of a typical case of the phantom measurements are shown in Fig. 2. The ^19^F 3D CSI scan of the 5.4 mM TFT solution within the body phantom led to a median SNR of 28 (range 25–29) in the optimal positioned voxel.

### Patients

Between July and December 2016, five patients with mCRC were included. The median age of patients was 63 years (range 31–66), and the mean body mass index was 23.8±3.9 (Table 1). All patients received TAS-102 as a single agent as a third- or fourth-line palliative systemic treatment. The dosage of TAS-102 was adjusted to the body surface area. Three patients received previous oncologic liver treatment including partial hepatectomy in two patients and radio-embolisation in one patient. Laboratory liver function test results at the start of TAS-102 treatment for all patients were < 5 times the upper limit of normal. Four patients had progressive disease after three cycles of TAS-102, and one patient after four cycles of TAS-102.

**Table 1:** Patient and treatment characteristics

	Patient 1	Patient 2	Patient 3	Patient 4	Patient 5
Age (years)	66	60	31	63	63
Sex	Female	Male	Female	Male	Male
BMI (kg/m^2^)	20.9	24.2	29.3	19.4	25.4
History of oncologic treatment of the liver	Hemihepatectomy	Radio-embolisation	Partial hepatectomy	None	None
TAS-102 dosage	50 mg b.i.d.	65 mg b.i.d.	55 mg b.i.d.	60 mg b.i.d.	70 mg b.i.d.
Cycle number	Cycle 2 day 8	Cycle 1 day 9	Cycle 1 day 9	Cycle 1 day 9	Cycle 1 day 9
Time of response evaluation according to RECIST	After 4 cycles TAS-102	After 3 cycles TAS-102	After 3 cycles TAS-102	After 3 cycles TAS-102	After 3 cycles TAS-102
Time to first disease progression (progression-free survival)	3.7 months	2.8 months	2.8 months	2.8 months	2.8 months
Time between oral intake of TAS-102 and first MRS examination	30 min	90 min	105 min	150 min	60 min
Timing of both localised CSI scans	14.24 min	14.24 min	14.24 min	14.24 min	14.24 min

Abbreviations: *BMI* body mass index, *b.i.d.* bi-daily, *CSI* chemical shift imaging, *min* minutes, *MRS* magnetic resonance spectroscopy, *RECIST* Response Evaluation Criteria In Solid Tumours, *TAS-102* trifluridine/tipiracil

### Measurements on patients

**Fig. 3:**
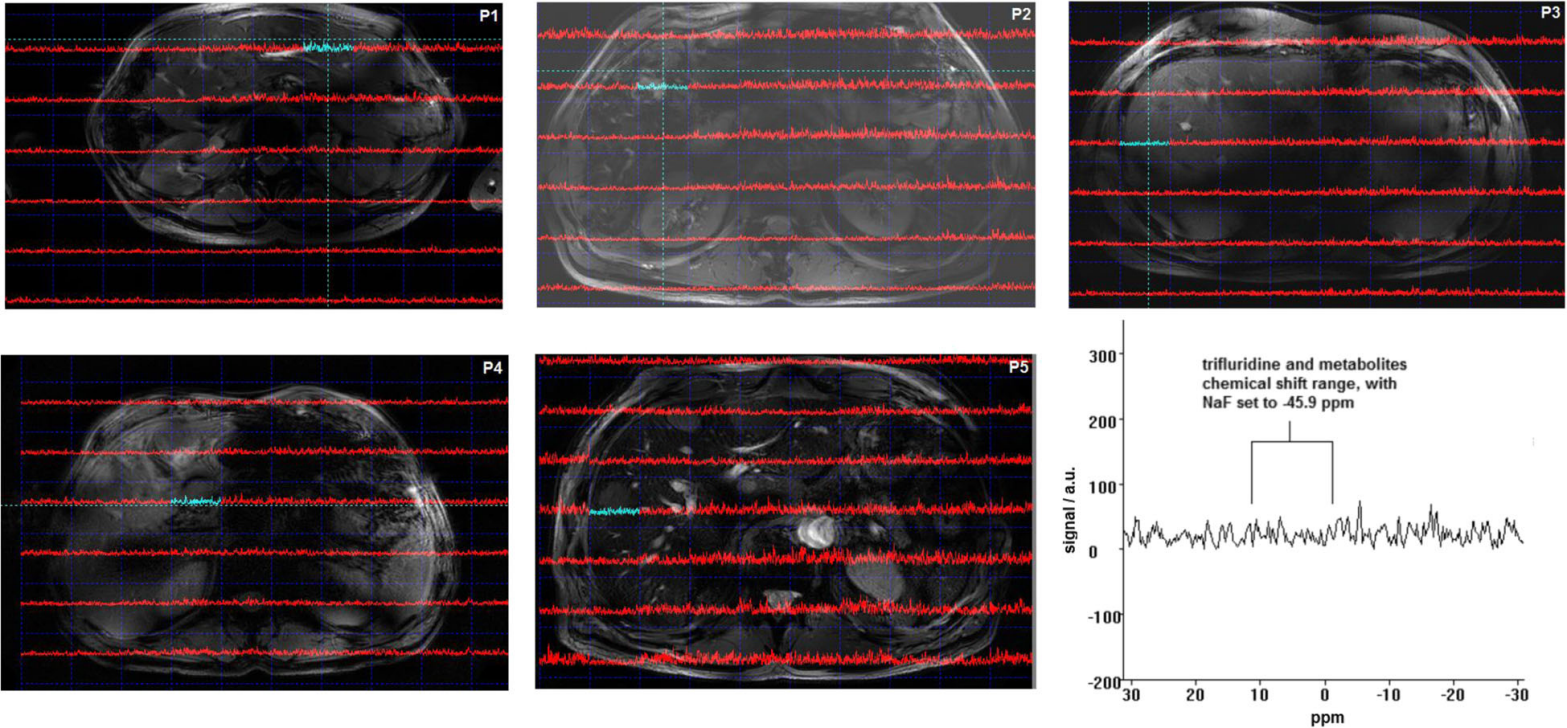
Patient measurements. Examples of ^19^F-MRS three-dimensional chemical shift imaging measurements in five metastatic colorectal cancer patients treated with TAS-102. The anatomical images of the regions of interest are shown, in this case the liver metastases, and an overlay of ^19^F spectra in *red* and *blue*. *Blue spectra* indicate the spectra measured in liver metastases, *red spectra* indicate the spectra measured in other liver tissue(s). In all images no distinct metabolic signals (SNR > 5.5 or > 4.8) were observed at around 10 ppm, where trifluridine should be visible. The spectra in this figure are shown in absolute mode. *P1–P5* patients 1–5

Examples of the ^19^F 3D CSI measurement of the five patients with mCRC are shown in Fig. 3. In all patients, during any of the CSI measurements, no significant signal (SNR > 5.5) was observed in any voxel in the TFT metabolite chemical shift range (median SNR 2.3, range 1.25–4.31). Also in the second more in-depth analysis, we did not find any spectra with signals that may have originated from the TAS-102 metabolites (median SNR 1.90, range 0.63–4.29).

## Discussion

This work demonstrated that, with the used setup, TAS-102 is not detectable during the early (< 3 h) post-administration phase in liver metastases of mCRC patients on a normal treatment schedule, despite the increased SNR during in vitro measurements when compared to capecitabine and metabolites. In the TAS-102 phantom, the SNR in 3D CSI mode was 1.3 times higher than the previous measured SNR of capecitabine and metabolites during in vitro measurements in unlocalised mode, when corrected for concentration difference and total scan time [10].

A possible explanation for not detecting TAS-102 in vivo using ^19^F-MRS may be the standard low dosage of TAS-102 administered in humans [2]. Clinical data on the distribution of TAS-102 after oral intake are only available for plasma; data on the distribution throughout the body are lacking. Previous in vivo murine studies detected TFT and its metabolites in the liver, but only after administering a much higher dosage of TAS-102 when compared to the routine dosage in humans (150–450 mg/kg in mice versus 35 mg/m^2^ in humans) and/or using a longer scanning time [14, 16]. Since the tissue volume in humans and thereby the volume of TAS-102 for the given dose is orders of magnitude larger than in mice, the sensitivity of ^19^F MRS at the given field strength should be higher in humans than in mice, even considering the larger receivers used. Also since previous measurements of capecitabine and metabolites using 7-T MRS and the currently used setup have shown significant signals in vivo [10], we assume this setup was sufficient to detect signals for concentrations of TFT and/or its metabolites equal to or greater than 0.45 mM. Typical pharmacokinetic parameters of TAS-102 in blood plasma after repeated administrations are roughly maximum 5 μg/ml [13, 17], which is equivalent to 0.017 mM and below the detection limit of 0.45 mM. This typical TAS-102 concentration of 5 μg/mL in blood plasma is comparable to the typical maximum plasma concentration of capecitabine and its metabolites 5'-deoxy-5-fluorouridine (5’DFUR) and FBAL, which are also roughly 5 μg/mL [20], but lead to higher concentrations in liver metastases of mCRC patients that are detectable with 7-T MRS [10]. Finally, we provided instructions for a light preceding breakfast and optimised the scanning timing to maximise available TFT and metabolite concentrations in the plasma and tissues of the included patients. It is therefore likely that the concentration of TAS-102 and metabolites remained low throughout the body and were therefore not detectable.

The following study limitations should be taken into account. The in vivo T1 and T2* values of TFT and its metabolites in liver and metastases are unknown. Our choices for the flip angle (15°) and repetition times of 25 and 50 ms correspond to the Ernst angle excitation when T1 is 0.72 s and 1.43 s respectively. A factor 4 shorter metabolite T1 of 0.18 s would lead to a factor 2 increase in SNR when applying the Ernst angle excitation and is likely not sufficient to enable the in vivo metabolite detection of TAS-102. Determination of T1 values in animal studies, using a much higher dosage of TAS-102 and with fewer limitations on the specific absorption rate, could give an indication of the T1 of TFT in human tissue and thereby possibly a more optimal choice of pulse sequence parameters. However, the choices of repetition time and excitation flip angle are constrained by technical limitations. For example, the use of shorter repetition times is limited by the acquisition duration, which should ideally be ≥ 1.25 T2*, and increasing the Ernst angle is limited by the available maximum B1 of the MR system (12 μT), which would lead to longer RF pulses with lower excitation bandwidth.

We included a small sample size of five patients. Three of them received previous oncologic liver treatment including surgery or radio-embolisation, which might have affected the TAS-102 uptake in liver metastases. Furthermore, all patients received various preceding systemic oncological treatments and had progressive disease at the first response evaluation (i.e. after three or four cycles of TAS-102), which might be an indication of poor TAS-102 uptake. We cannot exclude the notion that patients with a better treatment response or patients who are less pre-treated may have different pharmacokinetic parameters regarding TAS-102 uptake in the liver metastases. However, the question remains whether any possible increase in TAS-102 uptake in the liver metastases of these patients is large enough to increase the chance of detecting any clinically relevant TAS-102 signal using 7-T MRS. Finally, this study did not provide data on MRS TAS-102 metabolite detection during the late phase (> 3 h after TAS-102 administration). Therefore, we cannot conclude whether TAS-102 metabolites remain undetectable throughout the whole cycle of administration.

In conclusion, our study showed that it is not feasible to detect TAS-102 metabolites in the liver of mCRC patients using 7-T ^19^F-MRS with the current setup, when using an SNR threshold of > 4.8 and when TAS-102 is administered on a standard treatment schedule. Therefore, ^19^F MRS TAS-102 metabolite detection is not yet useful for the clinical early prediction of treatment response. As ^19^F-MRS is able to detect TAS-102 in phantom and murine models, ^19^F-MRS remains a potential tool to noninvasively detect metabolites and/or monitor metabolism when higher dosages of TAS-102 are administered, e.g. in organoid and animal studies.

## Abbreviations

3D: Three-dimensional
5-FU: 5-Fluorouracil
b.i.d: Bi-daily
BMI: Body mass index
CSI: Chemical shift imaging
FBAL: α-Fluoro-β-alanine
FUPA: α-Fluoro-ureidopropionic acid
IDL: Interactive data language
mCRC: Metastatic colorectal cancer
MR: Magnetic resonance
MRS: Magnetic resonance spectroscopy
MRSI: MRS imaging
RECIST: Response Evaluation Criteria In Solid Tumours
SNR: Signal-to-noise-ratio
TAS-102: Trifluridine/tipiracil
TFT: Trifluridine
